# Data on the anisotropic interfacial slip length over fibrous porous media

**DOI:** 10.1016/j.dib.2017.06.010

**Published:** 2017-06-10

**Authors:** Jingang Lu, Hye Kyeong Jang, Wook Ryol Hwang

**Affiliations:** School of Mechanical Engineering, Research Center for Aircraft Parts Technology (ReCAPT), Gyeongsang National University, Jinju 52828, Republic of Korea

## Abstract

To characterize the velocity slip at the fibrous porous media, data on the anisotropic slip length has been fully analyzed through direct simulations for various geometrical aspects of fiber architecture [2] ). This data article provides detailed slip lengths and slip coefficients in dimensionless form as a function of various geometrical parameters of fibrous porous media including the fiber volume fraction, the dimensionless void length and the normalized permeability. The data is presented for three different fiber packing structures (the quadrilateral, hexagonal and compressed hexagonal packing) in both the fiber and normal to fiber directions. Finally a set of curves from the complete fitted equation set are also provided in a single figure that can be used to estimate the slip length and the normalized permeability for a given fibrous porous media.

**Specifications Table**

**Table:** 

Subject area	Mechanical and Material Science Engineering
More specific subject area	Liquid composites molding, Fabrics/textiles
Type of data	Tables, graph/figure
How data was acquired	Direct numerical simulation
Data format	Raw and analyzed/processed
Experimental factors	Pressure-driven flows through a channel over a fibrous porous media were solved with various channel height and the data presented here is for the channel height larger than 10 times of fiber diameter, where the universal slip behavior is observed.
Experimental features	Dimensionless slip lengths and slip coefficients in the longitudinal (fiber) and transverse (normal to fiber) directions are presented as a function of various geometrical parameters of fibrous porous media including the fiber packing structure, the fiber volume fraction, the dimensionless void length and the normalized permeability.
Data source location	School of Mechanical Engineering, Research Center for Aircraft Parts Technology (ReCAPT), Gyeongsang National University, Jinju, Korea
Data accessibility	Data is within this article

**Value of the data**

• The data provided herein can be used to demonstrate the anisotropic interfacial slip in fibrous porous media in liquid composite molding.
• The data provided here can be used to obtain two important characteristic parameters for flows in fibrous porous media: the anisotropic normalized permeability and the anisotropic interfacial slip length (or slip coefficients).
• The data provided here can be used to construct the slip length tensor of the Navier-slip model, which replaces actual corrugated fibrous surfaces by an effective smooth boundary at the interface between fibrous porous media and fluid.
• Fig. 2 provided here may be used to estimate the dimensionless slip coefficient, which is the most important parameter in predicting the interfacial slip in fibrous porous media. For example, for a given fibrous porous media of a certain packing structure with fiber volume fraction, one may estimate the dimensionless slip length and the normalized permeability from Fig. 2.

## 1 Data

**Fig. 1:**
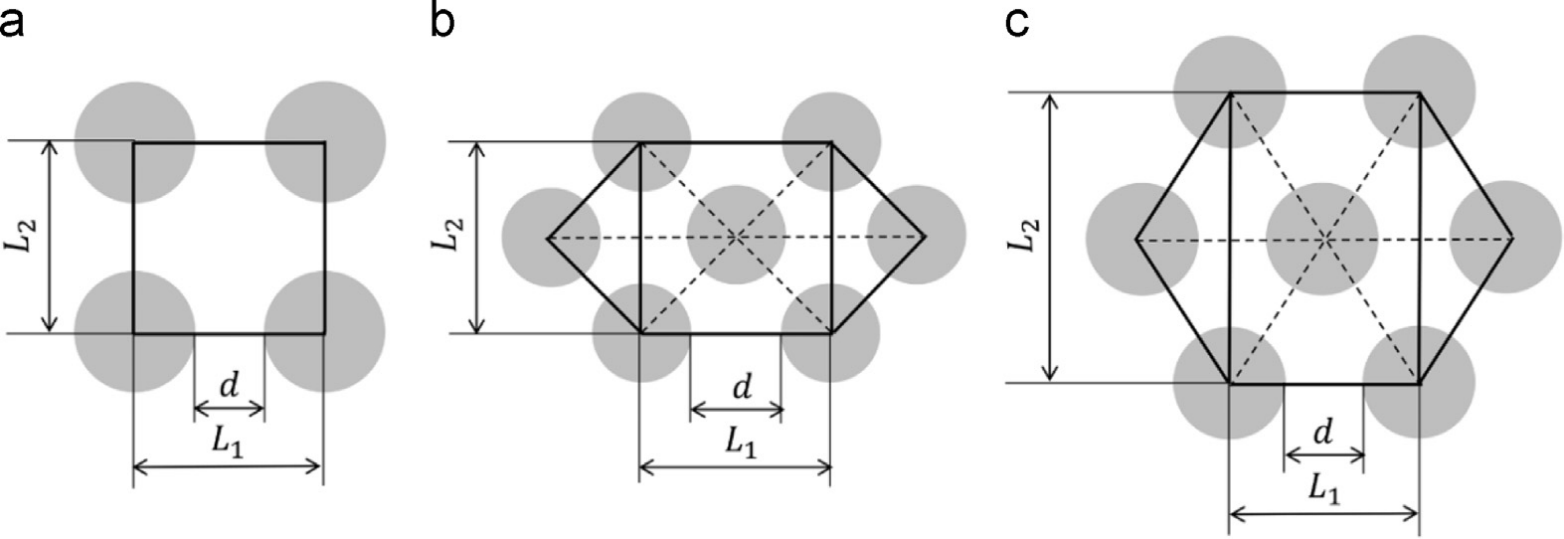
.Three different fiber packing structures of interest: (a) the quadrilateral packing (Quad); (b) the compressed hexagonal packing (Hex1); (c) the equilateral hexagonal packing (Hex2). Note that fiber volume fractions in all three arrangements are the same.

**Fig. 2:**
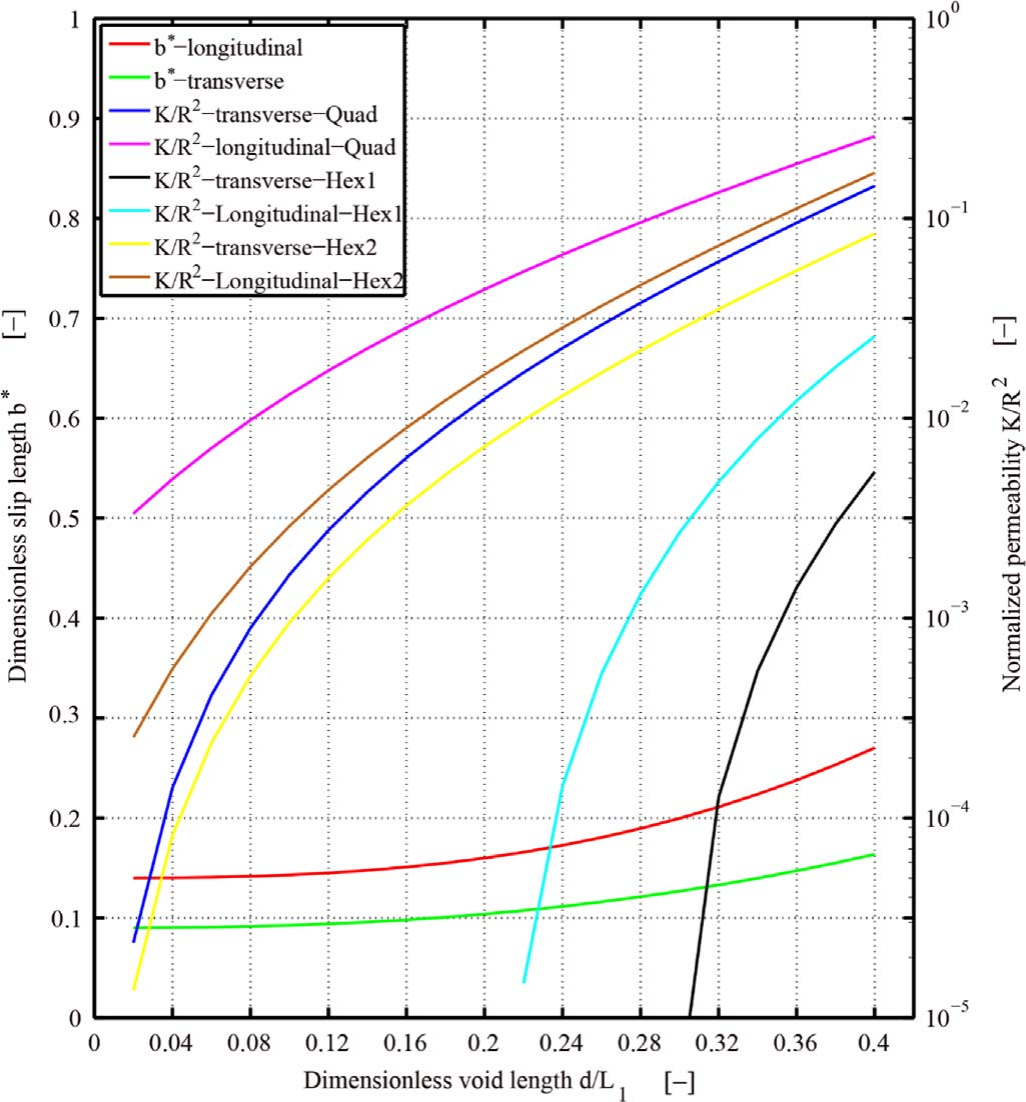
Fitted dimensionless slip lengths and normalized permeability as a function of dimensionless void length in transverse and longitudinal directions for various fiber packing structures.

Data in this article provides the dimensionless slip length $b^{*}$ and slip coefficient $\alpha_{BJ}$ as a function of the fiber volume fraction for three different representative fiber packing structures in both longitudinal and transverse directions of fibers (Fig. 1). The fiber volume fraction is the ratio of fiber volume to the volume of a unit cell in the fibrous porous media. The dimensionless slip length is defined as $b^{*}=b/R$ with the slip length b [m] and the fiber radius R [m]; and the dimensionless slip coefficient $\alpha_{BJ}=\beta \sqrt{K}$ with the slip coefficient β[m^−1^] of Beavers and Joseph [1] and the permeability K [m^2^]. For the quadrilateral packing structure, Table 1 is for the transverse direction and Table 2 is for the longitudinal direction. For the compressed hexagonal structure, Table 3, Table 4 contain slip length data for the transverse and longitudinal directions, respectively. Table 5, Table 6 list data on the slip length in each direction for the equilateral hexagonal packing structure. Table 7 describes the effect of the channel size on the slip length. In addition, we provided in each case the dimensionless void length $d^{*}$, which is the measure of fractional free slip area at the fluid/porous interface $d^{*}=d/L_{1}$ (Fig. 1), and the normalized permeability $K^{*}$. Plotted in Fig. 2 is the fitted dimensionless slip length and normalized permeability as a function of dimensionless void length in transverse and longitudinal directions for various fiber packing structures. Equation fitting is described in next section and in section 4.2 in Ref. [2].

**Table 1:** Slip length and slip coefficient over the quadrilateral packing structure in the transverse direction.

Volume fraction *V_f_*	Dimensionless void length d^*^	Normalized permeability K*	Dimensionless slip length b^*^	Dimensionless slip coefficient *α_BJ_*
0.15	0.563	5.77E−01	0.251	3.029
0.20	0.495	3.05E−01	0.209	2.638
0.25	0.436	1.73E−01	0.181	2.294
0.30	0.382	1.02E−01	0.161	1.984
0.35	0.332	6.07E−02	0.145	1.698
0.40	0.286	3.60E−02	0.132	1.434
0.45	0.243	2.10E−02	0.122	1.187
0.50	0.202	1.18E−02	0.113	0.959
0.55	0.163	6.23E−03	0.106	0.745
0.60	0.126	2.97E−03	0.100	0.546
0.65	0.090	1.19E−03	0.094	0.366
0.70	0.056	3.32E−04	0.089	0.204
0.75	0.023	3.28E−05	0.085	0.068

**Table 2:** Slip length and slip coefficient over the quadrilateral packing structure in the longitudinal direction.

Volume fraction *V_f_*	Dimensionless void length d^*^	Normalized permeability K*	Dimensionless slip length b^*^	Dimensionless slip coefficient *α_BJ_*
0.15	0.563	1.18E+00	0.450221	2.415
0.20	0.495	6.41E−01	0.361264	2.217
0.25	0.436	3.79E−01	0.303353	2.028
0.30	0.382	2.35E−01	0.262344	1.849
0.35	0.332	1.51E−01	0.23165	1.678
0.40	0.286	9.92E−02	0.207675	1.517
0.45	0.243	6.61E−02	0.188474	1.364
0.50	0.202	4.45E−02	0.172503	1.222
0.55	0.163	3.01E−02	0.15935	1.088
0.60	0.126	2.04E−02	0.148191	0.964
0.65	0.090	1.39E−02	0.138272	0.852
0.70	0.056	9.42E−03	0.129798	0.748
0.75	0.023	6.39E−03	0.122383	0.653

**Table 3:** Slip length and slip coefficient over the compressed hexagonal packing structure in the transverse direction.

Volume fraction *V_f_*	Dimensionless void length d^*^	Normalized permeability K*	Dimensionless slip length b^*^	Dimensionless slip coefficient *α_BJ_*
0.15	0.691	5.77E−01	0.402	1.891
0.20	0.643	3.05E−01	0.335	1.650
0.25	0.601	1.73E−01	0.290	1.435
0.30	0.563	1.02E−01	0.258	1.238
0.35	0.528	6.07E−02	0.233	1.057
0.40	0.495	3.60E−02	0.213	0.890
0.45	0.465	2.10E−02	0.197	0.735
0.50	0.436	1.18E−02	0.184	0.590
0.55	0.408	6.23E−03	0.172	0.458
0.60	0.382	2.97E−03	0.162	0.336
0.65	0.357	1.19E−03	0.154	0.224
0.70	0.332	3.32E−04	0.146	0.125
0.75	0.309	3.28E−05	0.139	0.041

**Table 4:** Slip length and slip coefficient over the compressed hexagonal packing structure in the longitudinal direction.

Volume fraction *V_f_*	Dimensionless void length d^*^	Normalized permeability K*	Dimensionless slip length b^*^	Dimensionless slip coefficient *α_BJ_*
0.15	0.691	1.18E+00	0.769	1.414
0.20	0.643	6.41E−01	0.624	1.283
0.25	0.601	3.79E−01	0.528	1.164
0.30	0.563	2.35E−01	0.460	1.055
0.35	0.528	1.51E−01	0.408	0.952
0.40	0.495	9.92E−02	0.368	0.856
0.45	0.465	6.61E−02	0.335	0.768
0.50	0.436	4.45E−02	0.308	0.685
0.55	0.408	3.01E−02	0.285	0.609
0.60	0.382	2.04E−02	0.265	0.538
0.65	0.357	1.39E−02	0.248	0.474
0.70	0.332	9.42E−03	0.234	0.415
0.75	0.309	6.39E−03	0.221	0.362

**Table 5:** Slip length and slip coefficient over the equilateral hexagonal packing structure in the transverse direction.

Volume fraction *V_f_*	Dimensionless void length d^*^	Normalized permeability K*	Dimensionless slip length b^*^	Dimensionless slip coefficient *α_BJ_*
0.15	0.593	5.73E−01	0.277	2.732
0.20	0.530	3.07E−01	0.231	2.398
0.25	0.475	1.78E−01	0.200	2.109
0.30	0.425	1.08E−01	0.178	1.852
0.35	0.379	6.73E−02	0.160	1.620
0.40	0.336	4.23E−02	0.146	1.405
0.45	0.296	2.65E−02	0.135	1.206
0.50	0.257	1.64E−02	0.126	1.021
0.55	0.221	9.95E−03	0.118	0.848
0.60	0.187	5.81E−03	0.111	0.690
0.65	0.153	3.20E−03	0.104	0.543
0.70	0.121	1.62E−03	0.099	0.406
0.75	0.091	7.12E−04	0.094	0.283
0.80	0.061	2.42E−04	0.090	0.173
0.85	0.032	4.48E−05	0.086	0.078

**Table 6:** Slip length and slip coefficient over the equilateral hexagonal packing structure in the longitudinal direction.

Volume fraction *V_f_*	Dimensionless void length d^*^	Normalized permeability K*	Dimensionless slip length b^*^	Dimensionless slip coefficient *α_BJ_*
0.15	0.593	1.15E+00	0.507	2.112
0.20	0.530	6.15E−01	0.407	1.925
0.25	0.475	3.58E−01	0.342	1.747
0.30	0.425	2.18E−01	0.296	1.576
0.35	0.379	1.36E−01	0.262	1.412
0.40	0.336	8.67E−02	0.235	1.255
0.45	0.296	5.54E−02	0.213	1.105
0.50	0.257	3.53E−02	0.195	0.962
0.55	0.221	2.22E−02	0.180	0.827
0.60	0.187	1.38E−02	0.168	0.701
0.65	0.153	8.34E−03	0.156	0.584
0.70	0.121	4.88E−03	0.147	0.475
0.75	0.091	2.73E−03	0.138	0.377
0.80	0.061	1.45E−03	0.131	0.290
0.85	0.032	7.23E−04	0.124	0.216

**Table 7:** Dimensionless slip lengths for three different fiber packing structures in both transverse and longitudinal directions as a function of the dimensionless channel height H/R$\left(V_{f}=0.5\right)$.

H/R	Quad		Hex1		Hex2	
	Transverse	Longitudinal	Transverse	Longitudinal	Transverse	Longitudinal
1	0.1872	0.2606	0.3973	0.7206	0.2173	0.3149
2	0.1478	0.2116	0.2834	0.4575	0.1684	0.2467
4	0.1287	0.1912	0.2257	0.3738	0.1444	0.2193
8	0.1206	0.1817	0.2022	0.3386	0.1342	0.2068
16	0.1168	0.1772	0.1919	0.3222	0.1296	0.2009
32	0.1150	0.1750	0.1872	0.3143	0.1273	0.1979
64	0.1140	0.1738	0.1849	0.3104	0.1263	0.1965
128	0.1135	0.1732	0.1838	0.3085	0.1257	0.1957

## 2 Experimental design, materials and methods

Pressure-driven channel flows between a no-slip wall on the top and a fibrous porous media on the bottom were solved to estimate the slip length and slip coefficient, which is the most important parameter in describing flows within the dual-scale porous media. The Navier–Stokes equation is solved for the two problems: one is the computational solution for the actual fiber arrangement on the bottom and the other is the analytical solution with the effective slip boundary condition on the bottom. The slip length and slip coefficient can be evaluated by comparison of the two solutions. Extensive numerical simulations were performed to obtain the slip coefficient in the longitudinal (fiber) and transverse (normal to fiber) directions are presented as a function of various geometrical parameters of fibrous porous media including the fiber packing structure, the fiber volume fraction, the dimensionless void length and the normalized permeability. By the mesh refinement study, the accuracy more than three significant digits were ensured in estimating the slip length and slip coefficient. The three different fiber packing structures are presented in Fig. 1 and data includes slip characterization from very low volume fraction of fibers (0.15) to highly packed cases (up to 0.75 for the quadrilateral and compressed hexagonal packings; 0.85 for the equilateral hexagonal packings). From Ref. [2], the slip length and slip coefficient can be conveniently expressed as a master curve based on the dimensionless void length, which is determined directly from the fiber volume fraction and the structure of the porous media, and the relationship is given here for the completeness:

(1) $$d^{*}\left(V_{f}\right)=\{\begin{array}{cc} 1-\sqrt{4V_{f}/\pi }, & \left(\text{Quadrilateral}\;\text{packing},\;\text{Quad}\right)\\ 1-\sqrt{2V_{f}/\pi }, & \left(\text{Compressed}\;\text{hexagonal}\;\text{packing},\;\text{Hex1}\right)\\ 1-\sqrt{2\sqrt{3}V_{f}/\pi }, & \left(\text{Equilateral}\;\text{hexagonal}\;\text{packing},\;\text{Hex2}\right) \end{array}.$$

As was discussed in Ref. [2] (Section 4.1), there is a dependence of the slip length and coefficients on the flow channel thickness H; however the dependence is removed completely for the channel height larger than 100 times of fiber radius R, where the universal slip behavior is observed independent of the channel size. All the data presented here is taken from the case H/R=128.

From the data on the slip length and slip coefficient presented in Table 1, Table 2, Table 3, Table 4, Table 5, Table 6 can be fitted in a universal way in a closed form, as a function of the dimensionless void length $d^{*}$ and the fitted equations in the longitudinal and the transverse direction is given as

(2) $$b_{∥}^{*}=1.56d^{{*}^{2.71}}+0.14,\;b_{⊥}^{*}=0.67d^{{*}^{2.41}}+0.09.$$

The normalized permeability in both directions can be fitted also as follows:

(3) $$K_{∥}^{*}=\{\begin{array}{cc} 0.162{V_{f}}^{0.845}{\left(1-V_{f}\right)}^{3}/V_{f}^{2} & \left(\text{Quad}/\text{Hex1}\right)\\ 0.095{V_{f}}^{0.426}{\left(1-V_{f}\right)}^{3}/V_{f}^{2} & \left(\mathrm{Hex2}\right) \end{array},K_{⊥}^{*}=\{\begin{array}{cc} 16/\left(9\pi \sqrt{2}\right){\left(\sqrt{\left(\pi /4\right)/V_{f}}-1\right)}^{5/2} & \left(\text{Quad}/\text{Hex1}\right)\\ 16/\left(9\pi \sqrt{6}\right){\left(\sqrt{\left(\pi /2\sqrt{3}\right)/V_{f}}-1\right)}^{5/2} & \left(\mathrm{Hex2}\right) \end{array}.$$

In the above equations, the symbols ′∥′ and ′⊥′ denote longitudinal and transverse directions, respectively and can be found in section 4.2 in Ref. [2]. Plots in Fig. 2 were constructed using the fitted form in Eqs. (1), (2), (3). The accuracy of the fitted equation can be found in Fig. 12 in Ref. [2] for the dimensionless slip coefficient, which can be calculated as $\alpha_{BJ}=\sqrt{K^{*}}/b^{*}$.
